# The effect of childhood socioeconomic status on depressive symptoms in middle-old age: the mediating role of life satisfaction

**DOI:** 10.1186/s12888-022-04046-3

**Published:** 2022-06-14

**Authors:** Lei Tang, Ruoyun Yin, Qian Hu, Zhaoya Fan, Fan Zhang

**Affiliations:** School of Public Health and Management, Research Center for Medicine and Social Development, Chongqing Medical University, No. 61 Daxuecheng Middle Road, Shapingba District, Chongqing, 400016 People’s Republic of China

**Keywords:** Childhood socioeconomic status, Depressive symptoms, Middle-old age, Mediating role, CHARLS

## Abstract

**Background:**

Some studies have shown that childhood socioeconomic status (SES) can influence the development and progression of depression in adulthood. This study aimed to analyze the effects of childhood SES on depressive symptoms in individuals of middle-old age and examine the potential mediating role of life satisfaction based on national data in China.

**Methods:**

Data were derived from the 2018 China Health and Retirement Longitudinal Study (CHARLS) data. A total of 17,129 individuals who were aged 45 years and older were included. The dependent variable was depressive symptoms in middle-old age. Childhood SES was the independent variable, and life satisfaction was the mediator. This study controlled four factors: general demographic characteristics, adult SES, childhood adversity and health and living status. Pearson correlations and hierarchical multiple regression analysis were performed, and the Baron and Kenny method was used to test the mediating role.

**Results:**

The score of depressive symptoms among all participants was 7.88 ± 6.65. Gender, age, adult SES, childhood adversity and health and living status all affected the development of depression symptom in middle-aged and elderly individuals in China. After adjusting for all covariates, the higher the childhood SES, the lower the depressive symptom score (*β* = − 0.422, *P* < 0.001). Life satisfaction had a partial mediating effect between childhood SES and depressive symptoms. Low childhood SES may improve life satisfaction (*β* = 0.051, *P* < 0.001) and life satisfaction indirectly decreases depressive symptom scores (*β* = − 0.403, *P* < 0.001).

**Conclusions:**

Life satisfaction was a partial mediator between childhood SES and depressive symptoms in middle and old age. Improving life satisfaction may serve as an intervention to reduce the incidence of depression symptoms in the future.

**Supplementary Information:**

The online version contains supplementary material available at 10.1186/s12888-022-04046-3.

## Background

Disease and social burden increase in the aging society. Psychological problems of the elderly have attracted more attention. Depression, which is a common problem affecting the mental health and quality of life of middle-aged people, affects an estimated 350 million individuals worldwide [1, 2]. Approximately 15% of adults aged 60 years and older suffer from a mental disorder, such as depression [3]. Depression is one of the most common mental disorders in old age. Depression increases the risk of cognitive dysfunction and senile dementia in the elderly and is a common risk factor for suicide [4, 5]. The aggravation of depression in middle-aged and elderly individual not only seriously affects their quality of life but also places a heavy burden on society and family. Depressive symptoms are the early signs of depression. Early detection of depressive symptoms is of great significance to prevent the development of the disease course and to provide treatment and intervention [6]. Some experts have suggested that health is affected by multiple factors, such as medical level (the number of local hospitals and medical staff, etc.), income, lifestyle, education and living environment [7]. Changes in the health status of middle-aged and elderly people are especially affected by various factors, such as depressive symptoms. Socioeconomic status (SES) has become one of the directions in medical research to explore the health of middle-aged and elderly people in recent years. SES is an important factor affecting the health of middle-aged and elderly individuals.

Socioeconomic status (SES) is a general measure of the working experience of an individual or a group of people and the economic and social status of an individual or a family relative to others. SES is a comprehensive reflection of the occupation, education level and economic income level of a group [8]. Studies have shown that low SES is associated with high psychiatric morbidity, such as the chronicity of depression [9]. The influence of childhood on the individual is a long and longitudinal cumulative process that occurs throughout life [10]. Childhood SES or childhood experiences may also affect individual health status in later life. Individuals with low SES in childhood may suffer from insufficient medical and health resources in middle and old age, leading to impaired health in middle and old age [11]. Survival and environmental problems in early childhood can lead to long-term changes in individual stress and have an adverse impact on mental health [12]. Some studies have shown that childhood SES can influence the development and progression of depression in adulthood, especially in middle-old age. Low childhood SES is associated with depressive symptoms in adulthood [13]. Poor childhood SES can increase the incidence of depression in middle and old age [14]. A study that followed depression for up to 15 years suggested that childhood SES influences depression through a series of economic stressors, limited social resources, and physical symptoms in adulthood [15]. A Japanese prospective cohort study also showed that low childhood SES has a long-latency effect on the onset of depression among older adults [16].

Studies have shown that improvement in life satisfaction of the elderly can significantly improve their mental health [17]. A longitudinal study of data from the Korean Welfare Panel Study (KWPS) showed that life satisfaction significantly increased with time in older adults, whereas depression decreased [18]. Middle-aged and elderly people with higher life satisfaction can live independently, take an active part in social activities, and have a higher sense of self-worth, thus promoting the development of their mental health. The life satisfaction of middle-aged and elderly people includes the satisfaction degree of all aspects of daily life, reflecting their daily living ability and life attitude. People with higher life satisfaction generally show more positive psychological states [19]. However, few studies have explored the mediating role of life satisfaction in the relationship between childhood SES and depressive symptoms.

Using CHARLS data, this study aims to investigate the status of Chinese depressive symptoms and life satisfaction in middle-old age, to analyze the effects of childhood SES on depressive symptoms and to explore the potential mediating role of life satisfaction between childhood SES and depressive symptoms in middle-old age. This research will add an empirical basis for the study on the impact of life satisfaction and SES on the health of middle-aged and elderly people and provide a scientific basis and intervention direction for further research on depression in middle-old age.

## Methods

### Sampling

As a cross-sectional study, the data were mainly derived from the 2018 wave of the China Health and Retirement Longitudinal Study (CHARLS). The CHARLS is an investigation of adults aged 45 or older from their families in China based on the Health and Retirement Study (HRS) using a stratified multistage PPS random sampling strategy [20]. The presurvey sample includes a total of 2685 people in 1570 households from 95 communities/villages in 32 counties/districts, and finally produces a set of high-quality data, demonstrating that it is feasible to conduct a health and old-age survey in China. The CHARLS conducted a national baseline survey in 2011–2012, successfully interviewing 17,708 individuals in 10,257 households. The respondents are generally representative of middle-aged and elderly people in China, covering 450 villages and residences in 150 counties and districts nationwide. The CHARLS cohort was followed up approximately every 2 years with wave 4 in 2018 representing the latest follow-up period. In addition, the CHARLS life history survey in 2014 retrospectively collected the life history information of all live respondents in the previous waves (2011 and 2013). The data include residence and relocation history, childhood history, and educational history. All methods used in this study were implemented in accordance with relevant CHARLS guidelines and regulations and the Declaration of Helsinki. All participants joined CHARLS voluntarily and signed a consent form before participation. Ethical approval for all the CHARLS waves was granted from the Institutional Review Board at Peking University. The IRB approval number for the main household survey, including anthropometrics, is IRB00001052–11015; the IRB approval number for biomarker collection was IRB00001052–11014.

In this study, the data of the CHARLS 2018 wave and 2014 life history data were mainly used. Middle-aged and elderly people ≥45 years old living in urban or rural areas were selected. In total, 20,452 people participated in the life history survey in 2014. Among them, 3165 were excluded due to death or loss of follow-up in the 2018 survey, 91 because they were younger than 45 years old, and 67 because they lived in special areas. Finally, 17,129 individuals were included in this study. For a robustness check, the missing values were handled using multiple imputation.

### Measures

#### Depressive symptoms in middle and old age

Depressive symptoms in middle and old age were assessed using the Center for Epidemiological Studies Depression Scale (CES-D) revised by Andersen (1994) [21]. The scale consists of 10 items, each of which is scored on a scale of 4, ranging from “Rarely or none of the time (<1 day)” to “Most or all of the time (5-7 days)” on a scale of 0–3, with Items 5 and 8 being scored in reverse. Studies have shown that this scale has a high reliability and validity in middle-aged and elderly people in China, and the total Cronbach’s α coefficient is 0.815. These results demonstrate that this method effectively measures the level of depressive symptoms in middle and old age [22]. The total score of the scale is between 0 and 30. The higher the score, the more severe the depressive symptoms.

#### Childhood socioeconomic status

Combined with the actual status of China and reference to the published literature on childhood SES, childhood SES in this study was comprehensively assessed based on the self-reported family economic status and educational level of female and male dependents [8, 23, 24]. A question was used to measure self-reported family economic status: When you were a child before age 17, compared to the average family in the same community/village at that time, how was your family’s financial situation? Self-reported family economic status was rated on a 5-point scale, with 1 for “A lot worse off than them”, 2 for “Somewhat worse off than them”, 3 for “Same as them”, 4 for “Somewhat better off than them”, and 5 for “A lot better off than them”. The educational level comprised five grades from illiteracy or semi-illiteracy (being able to read or write), primary school or home school, middle school, high school or vocational school, and university degree or above, which are recorded as 1–5 points. The value of each indicator was normalized as z-scores so that each component has a mean of 0 and a SD of 1. Finally, all the available z-scores of SES indicators were averaged to represent the score of childhood SES [25]. The higher the score was, the higher childhood SES.

#### Life satisfaction

Life satisfaction was measured using the question from the CHARLS 2018 wave questionnaire: “Please think about your life-as-a-whole. How satisfied are you with it?” The questions were asked on a five-point scale, with 0 for “Not at all satisfied”, 1 for “Not very satisfied”, 2 for “Somewhat satisfied”, 3 for “Very satisfied”, and 4 for “Completely satisfied”. Participants made choices according to their own circumstances.

#### Covariates

The current literature has demonstrated an association between adult SES and depression in old age. Poor control problems (such as smoking and alcohol use) may also be the result of an individual’s previous childhood conditions, and the path from childhood SES to mental health problems in old age may be related to sleep and alcohol [26, 27]. To demonstrate the independent influence of childhood SES on depression symptoms in middle and old age, factors from four aspects were controlled for general demographic characteristics, adult SES, childhood adversity and health and living status after reading and referring to relevant literature. The specific control factors were as follows: gender, age, residence, educational background, marital status, social interaction support, childhood parental quarrels, childhood parental violence, childhood famine, activities of daily living (ADL), sleep at night, and alcohol consumption (for details regarding each variable, see Table A in the Additional file 1).

### Statistical analyses

SPSS 25.0 was used for statistical analysis. Statistical significance was indicated by a two-tailed *P* < 0.05. The dependent variable was depressive symptoms in middle-old age. Childhood SES was the independent variable, and life satisfaction was the mediator. All covariates were categorical variables. Continuous variables are reported as the mean ± standard deviation (SD), and categorical variables are reported as percentages. As a robustness check, the multiple imputation method was considered to impute missing data by creating 20 imputed datasets. Pearson correlations and hierarchical multiple regression analysis were conducted to explore the relationship among childhood SES, life satisfaction and depressive symptoms in middle-old age.

Five models were conducted to test the effects. The first step was to construct a model that only included gender, age variables and childhood SES. In Models 2–4, variables, such as adult SES, child adversity, health and living status, were successively added for regression analysis. In Model 5, life satisfaction was added as a mediating variable for analysis. Multicollinearity was not considered as a problem if the variance inflation factor (VIF) value was less than 10 [28]. In this study, the VIF test revealed that the VIF values of the independent variable, mediating variable, and control variables were far less than 10.

The Baron and Kenny method was used to test mediation given that it is compatible with multiple imputation datasets [29]. The mediating effect can be determined by stepwise multiple regression analysis. The specific steps are as follows: 1) the regression effect of X on Y is (Y = *c*X + *e*_*1*_) and is significant, where *c* is the total effect of the independent variable X on the dependent variable Y; 2) the regression effect of X on M is M = *a*X + *e*_*2*_, and *a* is the effect of independent variable X on mediator variable M; 3) the regression effect of X and M on Y is (Y = *c’*X + *b*M + *e*_*3*_), *b* is the effect of the mediator variable M on the dependent variable Y after controlling the influence of the independent variable X, *c’* is the direct effect of the independent variable X on the dependent variable Y after controlling the influence of the mediator variable M, and e_1_, e_2_, and e_3_ represent the regression residual. If *b* is significant and *c’* is not significant, then M acts as a complete mediating effect. If *b* and *c* are both significant, then M has a partial mediating effect. If *b* is not significant, then additional tests will be required to verify the possible moderating or interactive effects of M in X and Y [30, 31]. The mediating effect is equal to the indirect effect, that is, the coefficient product *ab*. The relationship between the mediating effect and the total effect and the direct effect is as follows: *c* = *c’* + *ab* [32]. To understand the effect size of the mediating pathway, we calculated the proportion of the total effect of childhood SES on depressive symptoms in middle-old age that was mediated by life satisfaction using the formula *ab*/*c*. In this study, we hypothesized that life satisfaction (M) was the mediator of childhood SES (X) and depressive symptoms in middle-old age (Y), and the effect of childhood SES on depressive symptoms in middle-old age can be generated in whole or in part by life satisfaction.

## Results

### Descriptive statistics

Table 1 shows the descriptive statistics of the characteristics of the study population. A total of 17,129 samples were included from the CHARLS 2018 wave in this study. A total of 8022 (46.8%) were males, and 9107 (53.2%) were females. There were 6423 (37.5%) middle-aged people aged 45 to 60 years; 7784 (45.4%) young elderly people aged 60 to 75 years; and 2922 (17.1%) older people aged 75 years and over. In total, 3932 persons (23.0%) reported living in urban areas, whereas 13,197 persons (77.0%) reported living in rural areas. Illiterate or semiliterate participants comprised the largest group with 7637 participants (44.6%). Most respondents (84.8%) had a partner living with them. A total of 82.1% of the participants had complete data on depressive symptoms, and the depressive symptom score was 7.88 ± 6.65. The average life satisfaction score was 2.10 ± 0.96.

**Table 1:** The descriptive statistics of the characteristics of the study population (*N* = 17,129)

Variables		N (%)	Mean (SD)	Missing rate (%)
Depressive symptom		15,212 (82.1%)	7.88 (6.65)	17.9%
Childhood SES				
Mother’s education		16,411 (95.8%)	1.26 (0.72)	4.2%
Father’s education		15,870 (92.6%)	1.91 (1.17)	7.4%
Self-reported family economic status		17,029 (99.4%)	2.47 (0.98)	0.6%
Life satisfaction		17,129 (100%)	2.10 (0.96)	0.0%
Gender	Male	8022 (46.8%)		0.0%
	Female	9107 (53.2%)		
Age	45~	6423 (37.5%)		0.0%
	60~	7784 (45.4%)		
	75~	2922 (17.1%)		
Residence	Town	3932 (23.0%)		0.0%
	Rural	13,197 (77.0%)		
Education background	Illiterate or semi-illiterate	7637 (44.6%)		0.0%
	Primary school or home school	3849 (22.5%)		
	Middle school	3661 (21.4%)		
	High school or vocational school	1684 (9.8%)		
	University degree or above	298 (1.7%)		
Marital status	Have a spouse	14,525 (84.8%)		0.0%
	No spouse	2604 (15.2%)		
Social interaction support	Yes	8980 (52.4%)		0.1%
	No	8130 (47.5%)		
Childhood parental quarrels	Never	6847 (40.0%)		10.2%
	Rarely	4933 (28.8%)		
	Sometimes	2756 (16.1%)		
	Often	847 (4.9%)		
Childhood parental violence	Never	12,021 (70.2%)		10.9%
	Rarely	1969 (11.5%)		
	Sometimes	1008 (5.9%)		
	Often	270 (1.6%)		
Childhood famine	Yes	14,428 (84.2%)		0.0%
	No	2701 (15.8%)		
ADL	Yes	3746 (21.9%)		0.0%
	NO	13,383 (78.1%)		
Sleep at night	< 7 h	9637 (56.3%)		0.1%
	7 ~ 8	5826 (34.0%)		
	> 8 h	1652 (9.6%)		
Alcohol consumption	Yes	5665 (33.1%)		0.1%
	No	11,445 (66.8%)		

### Correlation analysis

As shown in Table 2, childhood SES, life satisfaction and depressive symptoms were correlated. Childhood SES was positively correlated with life satisfaction in middle and old age (*r* = 0.056, *P* < 0.01) and negatively correlated with depressive symptoms in middle and old age (*r* = − 0.116, *P* < 0.01). Life satisfaction in middle and old age was also negatively correlated with depressive symptoms in middle and old age (*r* = − 0.102, *P* < 0.01). The lower the childhood SES and life satisfaction scores in middle and old age, the higher the depressive symptom scores.

**Table 2:** Pearson correlations between childhood SES, life satisfaction and depressive symptoms

	Childhood SES	Life satisfaction	Depressive symptoms
Childhood SES	1	0.056**	−0.116**
Life satisfaction	–	1	−0.102**
Depressive symptoms	–	–	1

*Note*: ** indicates *P* < 0.01

### Hierarchical multiple regression analysis

Table 3 presents the results of hierarchical multiple regression analysis. Model 1 included childhood SES, and gender and age served as the only controls. Models 2–4 included, in turn, variables of adult socioeconomic status, childhood adverse experiences, adult health and living status. The results showed that the selected control variables of adulthood influenced depression symptoms in middle and old age. Adverse childhood experiences may have a greater impact on depression in older adults than childhood SES. After controlling for these factors, the Model 4 results showed that childhood SES still had a negative effect on depressive symptoms in middle and old age (*β* = − 0.422, 95% CI [− 0.576, − 0.267], *P* < 0.001). The lower the childhood SES, the higher the depression symptom score of middle and old age. Model 5 showed that life satisfaction significantly reduced the incidence of depressive symptoms (β = − 0.403, 95% CI [− 0.506, − 0.300], *P* < 0.001), and the effect of childhood SES on depressive symptoms was reduced with the inclusion of life satisfaction (*β* = − 0.401, 95% CI [− 0.555, − 0.247], *P* < 0.001). Thus, life satisfaction may play a mediating role in the relationship between depression symptoms in middle and old age and childhood SES. When incomplete data were imputed, the results were consistent (see Table B in the Additional file 1).

**Table 3:** Hierarchical multiple regression analysis: Effect of childhood SES and life satisfaction on depression symptoms

Variables		Model 1 Including sex and age	Model 2 Add variables of adult SES	Model 3 Add variables of childhood adversity	Model 4 Add variables of health and living status	Model 5 Add Life satisfaction
Childhood SES		−1.153*** (−1.300, −1.005)	−0.670*** (− 0.827, − 0.512)	−0.524*** (− 0.681, − 0.366)	−0.422*** (− 0.576, − 0.267)	−0.401*** (− 0.555, − 0.247)
Life satisfaction		–	–	–	–	− 0.403*** (− 0.506, − 0.300)
Gender (Male = 0)	Female	2.228*** (2.028, 2.427)	1.810*** (1.600, 2.020)	1.898*** (1.689, 2.108)	1.418*** (1.193, 1.642)	1.422*** (1.198, 1.647)
Age (45 ~ = 0)	60~	0.386*** (0.163, 0.609)	0.119 (−0.109, 0.346)	0.072 (−0.157, 0.300)	− 0.206 (− 0.429, 0.018)	−0.143 (− 0.366, 0.080)
	75~	−0.533*** (− 0.831, − 0.235)	−1.195*** (− 1.514, − 0.875)	−1.157*** (− 1.477, − 0.837)	−1.888*** (− 2.209, − 1.567)	−1.868*** (− 2.188, − 1.548)
Residence (Town = 0)	Rural	–	1.123*** (0.867, 1.379)	1.119*** (0.864, 1.374)	1.137*** (0.889, 1.386)	1.149*** (0.901, 1.398)
Education background (“Illiterate or semi-illiterate” = 0)	Primary school or home school	–	−0.553*** (− 0.820, − 0.286)	−0.552*** (− 0.818, − 0.287)	−0.310* (− 0.570, − 0.050)	−0.315* (− 0.575, − 0.056)
	Middle school	–	−1.175*** (− 1.464, − 0.886)	−1.172*** (− 1.460, − 0.884)	−0.897*** (− 1.179, − 0.616)	−0.908*** (− 1.189, − 0.626)
	High school or vocational school	–	−1.652*** (− 2.038, − 1.265)	−1.612*** (− 1.998, − 1.227)	−1.384*** (− 1.761, − 1.007)	−1.409*** (− 1.785, − 1.032)
	University degree or above	–	−1.831*** (− 2.641, − 1.021)	−1.839*** (− 2.646, − 1.033)	−1.402*** (− 2.188, − 0.616)	−1.411*** (− 2.196, − 0.626)
Marital status (“No spouse” = 0)	Have a spouse	–	− 0.962*** (− 1.258, − 0.667)	−0.966*** (− 1.259, − 0.673)	−0.734*** (− 1.020, − 0.448)	−0.672*** (− 0.958, − 0.386)
Social interaction support (No = 0)	Yes	–	− 0.398*** (− 0.602, − 0.194)	− 0.468*** (− 0.671, − 0.265)	−0.238* (− 0.437, − 0.039)	−0.206* (− 0.405, − 0.007)
Childhood parental quarrels (Never = 0)	Rarely	–	–	0.318* (0.069, 0.567)	0.295* (0.050, 0.539)	0.270* (0.026, 0.514)
	Sometimes	–	–	0.708*** (0.387, 1.029)	0.670*** (0.357, 0.984)	0.632*** (0.319, 0.946)
	Often	–	–	0.850** (0.310, 1.390)	0.802** (0.281, 1.322)	0.735** (0.216, 1.255)
Childhood parental violence (Never = 0)	Rarely	–	–	0.739*** (0.413, 1.066)	0.677*** (0.360, 0.994)	0.665*** (0.349, 0.981)
	Sometimes	–	–	1.137*** (0.670, 1.603)	0.996*** (0.547, 1.446)	0.964*** (0.516, 1.413)
	Often	–	–	1.731*** (0.830, 2.632)	1.617*** (0.747, 2.486)	1.589*** (0.720, 2.458)
Childhood famine (No = 0)	Yes	–	–	1.272*** (0.993, 1.550)	1.112*** (0.841, 1.383)	1.122*** (0.851, 1.392)
ADL (No = 0)	Yes	–	–		2.248*** (1.995, 2.502)	2.073*** (1.816, 2.330)
Sleep at night (“< 7 h” = 0)	7 ~ 8 h	–	–	–	− 2.405*** (−2.614, −2.196)	−2.374*** (− 2.583, − 2.165)
	> 8 h	–	–	–	− 2.544*** (− 2.880, − 2.208)	−2.537*** (− 2.873, − 2.201)
Alcohol consumption (No = 0)	Yes	–	–	–	−0.505*** (−0.733, −0.276)	−0.484*** (− 0.712, − 0.256)

*Note*: * indicates *P* < 0.05, ** indicates *P* < 0.01, *** indicates *P* < 0.001

### Mediating effect analysis

Table 4 shows the results of the mediation analysis. All covariates were controlled, and multivariate stepwise regression analyses were performed. First, childhood SES had a negative predictive effect on depressive symptoms in middle and old age (*β* = − 0.422, *P* < 0.001). Second, childhood SES was divided into independent variables, and life satisfaction was divided into dependent variables. Linear regression analysis was performed. The results showed that childhood SES had a positive predictive effect on life satisfaction (*β* = 0.051, *P* < 0.001). Finally, childhood SES and life satisfaction were used as independent variables, and the depressive symptom score was used as a dependent variable for regression analysis. The effects of childhood SES and life satisfaction on depressive symptoms in middle and old age were significant. Life satisfaction played a partial mediating role in childhood SES and depressive symptoms in middle and old age, and low childhood SES may indirectly decrease the occurrence of depression by increasing life satisfaction. The ratio of the mediating effect was *ab*/*c*, where *c* was the total effect of X on Y (*c* = − 0.422), and *ab* was the mediating effect of the intermediate variable M (*ab* = 0.051*-0.403). Therefore, the ratio of the mediating effect to the total effect was *ab*/*c* = 4.87%.

**Table 4:** The results of the mediation analysis

Step	Dependent Variables	Independence (Mediating) Factor	*B*	S.E.	*t*	*P*
1	Depressive symptom (Y)	Childhood SES (X)	−0.422	0.079	−5.353	< 0.001
2	Life satisfaction (M)	Childhood SES (X)	0.051	0.011	4.400	< 0.001
3	Depressive symptom (Y)	Childhood SES (X)	−0.401	0.079	−5.102	< 0.001
		Life satisfaction (M)	−0.403	0.052	−7.693	< 0.001

*Note*: gender, age, residence, educational background, marital status, social interaction support; childhood parental quarrels, childhood parental violence, childhood famine; ADL, sleep at night, alcohol consumption were covariates

## Discussion

Data from CHARLS can roughly represent middle-aged and elderly people in China, covering 450 villages and residences in 150 counties and districts nationwide [20]. This study used data from the CHARLS in the 2018 wave, assessed the status of depression and life satisfaction in middle-old age in China, analyzed the effect of the childhood SES on depression after controlling for some factors, and explored the mediating role of life satisfaction between childhood SES and depression. However, the average score of depressive symptoms or the incidence of depression varies from study to study due to aging, cohort differences, or changes in sample composition, which may lead to a bias in the estimated associations. Results from the CHARLS studies on depressive symptoms from different years were compared. The results showed that the score of depressive symptoms in middle and old age was 7.88 ± 6.65 in CHARLS in 2018, and multiple linear regression showed that gender, age, residence, education level and marital status all affected the development of depression symptom in middle-aged and elderly individuals in China. The depression symptom of females, middle-aged people aged 45–59 years, rural residents and those without a spouse was higher than that of males, elderly people over 75 years, urban residents, and those with spouses, respectively. With the increase in education, depression levels tend to decrease. This result was consistent with the analysis results of CHARLS data in 2013 and 2015 [33–35]. Lower SES in childhood was associated with a higher the score of depressive symptoms [36, 37].

Previous studies indicated that childhood adversity and health and living status significantly impacted the health of middle-aged and elderly people [38, 39]. In this study, multivariate regression analysis showed significant differences in childhood adversity and health and living status, such as childhood parental violence, ADL, and alcohol consumption. Middle-aged and older adults with impaired ADL are at significantly increased risk of depression [40, 41]. Experiencing more adverse events in childhood was associated with an increased risk of depression in adulthood [42, 43]. This study showed a significant association between childhood parental quarrels and the incidence of depression. In addition, parents fighting each other can produce long-lasting and profound effects on children, which has been the focus of scholars’ attention [44]. This study also showed that alcohol consumption was a protective factor for depression in middle-aged and older adults. However, foreign studies have shown that alcohol consumption can lead to depression [26]. This difference may be related to the cultural differences between China and the West. Most people in China drink occasionally to be social and promote sleep.

Most studies have shown a significant relationship between childhood SES and later life health [45, 46]. This study showed that after controlling for the above factors, the influence of childhood SES on depression in middle and old age remained significant. A study has the similar results to a study that used data from the Survey on Health, Aging, and Retirement in Europe (SHARE), which is a sister survey of CHARLS. Its results showed that childhood SES was positively associated with late-adulthood mental health and that the link was particularly strong for women [27]. Some studies have shown that the risk of depression in middle and old age in individuals with poor childhood SES is twice as high as that in individuals with good childhood SES [47]. People with low SES experience more setbacks and difficulties at work and in life, which subsequently worsens their mental health. Healthy and successful individuals with low SES will strive to join the high SES group, whereas there will be people in the high SES group who have fallen to low SES for various reasons [48]. Childhood development of psychological and health-related behaviors, such as stress and anxiety, can affect health-related behaviors in middle and old age and increase the risk of mental illness in middle and old age [49]. Low childhood SES may lead to cumulative disadvantages, such as poor nutrition and health in childhood and the lack of good educational opportunities. Compared with others, these individuals are more likely to have negative emotions, which will further affect future life decisions and even lead to low adult SES. The lower the adult SES, the higher the risk of depression [50].

Life satisfaction is significantly negatively correlated with the occurrence of depression, which is consistent with the results of previous studies [51, 52]. The results of this study showed the partial mediating effect of life satisfaction on the effect of childhood SES on depression in middle and old age. People with low childhood SES may indirectly affect the occurrence of depression by increasing life satisfaction. Life satisfaction is a relatively stable and lasting attitude of the elderly that reflects a satisfactory degree in all aspects of life. Life satisfaction more closely reflects the psychological dimension of middle-aged and elderly people and focuses on subjective well-being [53]. SES is one of the predictors of life satisfaction in middle-aged and elderly people [54]. A previous meta-analysis showed that higher SES is associated with greater life satisfaction among older adults [55]. Life satisfaction is often used as a measure of quality of life. Compared with objective social indicators, life satisfaction provides a more complex understanding of one’s living environment, considering people’s different reactions, interpretations, and adjustments to external social conditions [56]. Life satisfaction can also represent a more stable and diversified measure of the lives of middle-aged and elderly people [55]. Higher adult SES, support for family members and other aspects of life may increase the life satisfaction of middle-aged and elderly people, moderate the adverse effects of childhood, and thus improve their physical and psychological status [57, 58]. Studies have shown that life satisfaction is particularly strongly associated with health [59]. Health literacy, health status, and ADL were associated with life satisfaction in older age individuals [60]. People who are dissatisfied with their lives are 41 times more likely to have depressive symptoms than people who are satisfied with their lives [61]. Life satisfaction is associated with positive health behaviors and better physical and mental health outcomes [62]. Life satisfaction may compensate for the psychological trauma caused by poor childhood SES and create a positive and beautiful living environment, thus reducing the occurrence of depressive symptoms.

Some foreign studies have explored the mediating effect of adult education or adult SES on childhood SES and depression, but the results of the reports are contradictory [37, 63]. The reasons for the differences may result from the differing importance of adult SES and health in each country as well as differences in the age groups assessed for depression. Depression is influenced by multiple factors, making it highly unlikely that any single factor would completely explain an association with an independent variable [64]. However, we cannot ignore the positive effects of life satisfaction on depressive symptoms in middle-aged and older adults. Data from the 2016 Chinese family group study (CFPS) survey, which is a typical family longitudinal survey in China, showed that life satisfaction completely mediated the relationship between social capital and depressive symptoms [65]. Most studies have also shown that life satisfaction, as a type of positive psychological status, can reduce depressive symptoms [66, 67]. This finding was also confirmed by a partial mediating effect of life satisfaction. Life satisfaction can moderate the effect of childhood SES on the risk of depression. In the future, improving life satisfaction may serve as a potential intervention to reduce the incidence of depression symptoms, improve the mental health of middle-aged and elderly people, and promote healthy aging.

Although the present study has many merits, it is not without limitations. As a large cohort study, CHARLS included data that were exclusively self-reported by the respondents. Although professional investigators conducted the survey, the existence of report bias could not be excluded. Cohort studies of middle-aged and elderly people also cannot ignore the effect of survival, which was not discussed in this study. However, there is no denying the value of CHARLS as the largest cohort study of middle-aged and elderly populations in China. Using the latest wave of data, this cross-sectional study was designed to analyze associations between childhood SES, life satisfaction, and depressive symptoms in middle and old age, but causality was not assessed. In the future, we can continue to use the database to study the changing trend of depression symptoms over the years and the dynamic influence of various factors on the occurrence and development of depression in middle-aged and elderly people in China and to explore the causal association and other potential mutual influences. The hope is to help improve the mental health and quality of life of the elderly in an aging society.

## Conclusion

This study showed that life satisfaction and childhood SES were negatively correlated with depressive symptoms in individuals of middle-old age. Life satisfaction was a partial mediator between childhood SES and depression symptoms. Improving life satisfaction may serve as an intervention to reduce the incidence of depression symptoms in the future.

## Supplementary Information

**Additional file 1: Table A.** Details of variables covered in this article. **Table B.** Hierarchical multiple regression analysis: Effect of childhood SES and life satisfaction on depression symptoms include missing data.

## Data Availability

The datasets analysed during the current study are available from the CHARLS repository [http://charls.pku.edu.cn].

## Abbreviations

SES: Socioeconomic status
CHARLS: China Health and Retirement Longitudinal Study
CES-D: The Center for Epidemiological Studies Depression Scale
ADL: Activities of daily living
SD: Standard deviation
VIF: Variance inflation factor
