# Supplementary material for: The effect of childhood socioeconomic status on depressive symptoms in middle-old age: the mediating role of life satisfaction
Source: BMC Psychiatry. 2022 Jun 14;22:398. doi: 10.1186/s12888-022-04046-3 (PMC9195317; doi:10.1186/s12888-022-04046-3)
Supplement: Supplementary file 1 — Additional file 1: Table A. Details of variables covered in this article. Table B. Hierarchical multiple regression analysis: Effect of childhood SES and life satisfaction on depression symptoms include missing data. [file 12888_2022_4046_MOESM1_ESM.docx]

**Additional file**

| **Table A. Details of variables covered in this article.** | |
| --- | --- |
| **Included variables** | **The corresponding problem in CHARLS 2018 wave or 2014 life history and A standard of classification or measurement** |
| Depression in middle and old age | (1) I was bothered by things that don’t usually bother me; (2) I had trouble keeping my mind on what I was doing; (3) I felt depressed; (4) I felt everything I did was an effort; (5) I felt hopeful about the future; (6) I felt fearful; (7) My sleep was restless; (8) I was happy; (9) I felt lonely; (10) I could not get “going”.  0 for "Rarely or none of the time (<1 day)"; 1 for "Some or a little of the time"; 2 for "Occasionally or a moderate amount of the time"; 3 for "Most or all of the time" |
| Childhood socioeconomic status | - Childhood SES in the study was measured before the age of 17. - The educational level includes five grades from illiteracy or semi-illiteracy (be able to read or write), primary school or home school, middle school, high school or vocational school, university degree or above, which are recorded as 1-5 points. - Self-reported family financial conditions: “When you were a child before age 17, compared to the average family in the same community/village at that time, how was your family’s financial situation?” It was a 5-point scale, with 1 for " A lot worse off than them ", 2 for " Somewhat worse off than them ", 3 for " Same as them ", 4 for " Somewhat better off than them ", and 5 for " A lot better off than them ". - The total score is the sum of all the scores. |
| Life satisfaction | “Please think about your life-as-a-whole. How satisfied are you with it?”  0 for " Not at all satisfied ", 1 for " Not very satisfied ", 2 for " Somewhat satisfied ", 3 for " Very satisfied ", and 4 for " Completely satisfied " |
| Gender | “Interviewer record the Respondent’s gender”  Male or Female |
| Age | “What’s your actual date of birth?”  Calculate the age; This study was based on the new age segmentation proposed by the United Nations World Health Organization, in which middle-aged people aged 45 to 59, young elderly people aged 60 to 74 and older people aged 75 and above.  So, the three age groups are as follows: 45-59, 60-74, 75 and above. |
| Residence | “Was your address, BB000_W3, in the village or city/town?”  Town or Rural |
| Educational background | “What’s the highest level of education your have now (not including adult education)? What is the highest adult schooling degree or diploma you have?”  Illiterate or semi-illiterate; Primary school or home school; Middle school; High school or vocational school; University degree or above |
| Marital status | “What is your marital status?”  “Have a spouse” or “No spouse” |
| social interaction support | “Have you done any of these activities in the last month? (Check all that apply)”  Yes or No |
| childhood parental quarrels | “Did your parents often quarrel?”  “Never”, “Rarely”, “Sometimes”, “Often” |
| childhood parental violence | “Have your father ever beat up your mother? Have your mother ever beat up your father?”  “Never”, “Rarely”, “Sometimes”, “Often” |
| childhood famine | “When you were a child before age 17 was there ever a time when your family did not have enough food to eat? Between 1958-1962 did you and your family (including your grandparents, parents, siblings, children and so on) experience starvation?”  Yes or No |
| Activities of daily living (ADL) | **ADL have BADL and IADL, with 12 questions in total**. Each question has four options as follows: “No, I don’t have any difficulty”, “I have difficulty but can still do it”, “Yes, I have difficulty and need help”, “I can not do it”. Those who answer the first two options are deemed to have normal function, while those who answer the last two options are deemed to have disabled function. As long as the function of one of these activities is lost, BADL or IADL is considered impaired. Damage of BADL or IADL is considered impaired ADL [1-3].  **The BADL measure has six specific problems as follows –**  (1) Because of health and memory problems, do you have any difficulty with **dressing**? Dressing includes taking clothes out from a closet, putting them on, buttoning up, and fastening a belt.  (2) Because of health and memory problems, do you have any difficulty with **bathing or showering**?  (3) Because of health and memory problems, do you have any difficulty with **eating**, such as cutting up your food? (Definition: By eating, we mean eating food by oneself when it is ready)  (4) Do you have any difficulty with **getting into or out of bed**?  (5) Because of health and memory problems, do you have any difficulties with **using the toilet**, including getting up and down?  (6) Because of health and memory problems, do you have any difficulties with **controlling urination and defecation**? If you use a catheter (conduit) or a pouch by yourself, then you are not considered to have difficulties.  **The IADL measure has six specific problems as follows –**  (1) Because of health and memory problems, do you have any difficulties with **doing household chores**? (Definition: By doing household chores, we mean house cleaning, doing dishes, making the bed, and arranging the house).  (2) Because of health and memory problems, do you have any difficulties with **preparing hot meals**? (Definition: By preparing hot meals, we mean preparing ingredients, cooking, and serving food).  (3) Because of health and memory problems, do you have any difficulties with **shopping for groceries**? By shopping, we mean deciding what to buy and paying for it.  (4) Because of health and memory problems, do you have any difficulties with **making phone calls**?  (5) Because of health and memory problems, do you have any difficulties with **taking medications**? By taking medications, we mean taking the right portion of medication right on time.  (6) Because of health and memory problems, do you have any difficulties with **managing your money**, such as paying your bills, keeping track of expenses, or managing assets? |
| sleep at night | “During the past month, how many hours of actual sleep did you get at night (average hours for one night)? (This may be shorter than the number of hours you spend in bed.)”  According to the National Sleep Foundation, the normal sleep time for middle-aged and elderly people is 7 to 8 hours. In this study, the sleep time was divided into short sleep group (< 7h), normal sleep group (7~h) and long sleep group (> 8h). |
| alcohol consumption | “Did you drink any alcoholic beverages, such as beer, wine, or liquor in the past year? How often?”  1. Drink more than once a month; 2. Drink but less than once a month; 3. None of these  If the participant selects "1" or "2", it means "drinking" (Yes), and if the participant selects "3", it means "not drinking" (No). |

[1] Katz, S., 1983. Assessing self-maintenance: activities of daily living, mobility, and instrumental activities of daily living. J Am Geriatr Soc 31 (12), 721–727. https://doi.org/10.1111/j.1532-5415.1983.tb03391.x.

[2] Katz, S., Ford, A.B., Moskowitz, R.W., Jackson, B.A., Jaffe, M.W., 1963. Studies of Illness in the Aged. The Index of Adl: A Standardized Measure of Biological and Psychosocial Function. Jama 185, 914–919. https://doi. org/10.1001/jama.1963.03060120024016.

[3] Liu N, Cadilhac DA, Kilkenny MF, Liang Y. Changes in the prevalence of chronic disability in China: evidence from the China Health and Retirement Longitudinal Study. Public Health. 2020;185:102-109. doi: 10.1016/j.puhe.2020.03.032.

[4] Chen JF, Fang MW, Xiao CH, Ma X. Activities of Daily Living and Depressive Symptoms in the Chinese Elderly. Chinese General Practice. 2020;23(22):2852-2855.

| **Table B. Hierarchical multiple regression analysis: Effect of childhood SES and life satisfaction on depression symptoms include missing data.** | | | | | | |
| --- | --- | --- | --- | --- | --- | --- |
| Variables | | Model 1  Including sex and age | Model 2  Add variables of adult SES | Model 3  Add variables of childhood adversity | Model 4  Add variables of health and living status | Model 5  Add Life satisfaction |
| Childhood SES | | -1.067***  (-1.223, -0.911) | -0.591***  (-0.757, -0.424) | -0.483***  (-0.220, -0.101) | -0.380***  (-0.543, -0.218) | -0.363***  (-0.526, -0.201) |
| Life satisfaction | | - | - | - | - | -0.367***  (-0.479, -0.254) |
| Gender (Male=0) | Female | 2.125***  (1.907, 2.342) | 1.748***  (1.521, 1.976) | 1.823***  (1.596, 2.051) | 1.364***  (1.120, 1.609) | 1.371***  (1.127, 1.616) |
| Age (45~ = 0) | 60~ | 0.272*  (0.032, 0.511) | 0.052  (-0.192, 0.295) | 0.041  (-0.204, 0.285) | -0.208  (-0.447, 0.031) | -0.149  (-0.388, 0.091) |
|  | 75~ | -1.016***  (-1.351, -0.680) | -1.580***  (-1.935, -1.224) | -1.491***  (-1.848, -1.134) | -2.157***  (-2.514, -1.801) | -2.155***  (-2.511, -1.798) |
| Residence (Town = 0) | Rural | - | 1.152***  (0.879, 1.425) | 1.143***  (0.871, 1.415) | 1.175***  (0.910, 1.440) | 1.185***  (0.921, 1.450) |
| Education background (“Illiterate or semi-illiterate” = 0) | Primary school or home school | - | -0.512***  (-0.803, -0.221) | -0.529***  (-0.819, -0.240) | -0.309*  (-0.593, -0.026) | -0.307*  (-0.590, -0.024) |
|  | Middle school | - | -1.057***  (-1.366, -0.748) | -1.072***  (-1.379, -0.764) | -0.826***  (-1.127, -0.525) | -0.828***  (-1.129, -0.527) |
|  | High school or vocational school | - | -1.544***  (-1.950, -1.137) | -1.511***  (-1.916, -1.106) | -1.306***  (-1.702, -0.910) | -1.323***  (-1.718, -0.927) |
|  | University degree or above | - | -1.803***  (-2.636, -0.970) | -1.843***  (-2.671, -1.015) | -1.455***  (-2.262, -0.647) | -1.452***  (-2.259, -0.645) |
| Marital status (“No spouse” = 0) | Have a spouse | - | -1.037***  (-1.370, -0.704) | -1.065***  (-1.396, -0.734) | -0.843***  (-1.165, -0.520) | -0.776***  (-1.099, -0.453) |
| Social interaction support (No = 0) | Yes | - | -0.323**  (-0.546, -0.101) | -0.395***  (-0.617, -0.174) | -0.176  (-0.394, 0.041) | -0.147  (-0.364, -0.070) |
| Childhood parental quarrels (Never = 0) | Rarely | - | - | 0.379**  (0.118, 0.640) | 0.393**  (0.139, 0.648) | 0.381**  (0.126, 0.635) |
|  | Sometimes | - | - | 0.736***  (0.400, 1.071) | 0.713***  (0.386, 1.040) | 0.686***  (0.359, 1.012) |
|  | Often | - | - | 0.790**  (0.199, 1.382) | 0.776**  (0.200, 1.352) | 0.718*  (0.143, 1.294) |
| Childhood parental violence (Never = 0) | Rarely | - | - | 0.769***  (0.419, 1.120) | 0.737***  (0.396, 1.078) | 0.731***  (0.391, 1.072) |
|  | Sometimes | - | - | 1.274***  (0.783, 1.765) | 1.153***  (0.675, 1.631) | 1.129***  (0.651, 1.606) |
|  | Often | - | - | 1.846***  (0.887, 2.805) | 1.749***  (0.815, 2.683) | 1.724***  (0.792, 2.657) |
| Childhood famine (No = 0) | Yes | - | - | 1.118***  (0.815, 1.422) | 0.971***  (0.675, 1.267) | 0.980***  (0.684, 1.275) |
| ADL (No = 0) | Yes | - | - |  | 2.257***  (1.975, 2.539) | 2.086***  (1.800, 2.373) |
| Sleep at night (“<7h” = 0) | 7~8 | - | - |  | -2.261***  (-2.487, -2.035) | -2.231***  (-2.457, -2.005) |
|  | >8h | - | - |  | -2.616***  (-2.990, -2.242) | -2.615***  (-2.988, -2.241) |
| Alcohol consumption (No = 0) | Yes | - | - |  | -0.492**  (-0.739, -0.245) | -0.470**  (-0.716, -0.223) |

Note: * indicates P < 0.05, ** indicates P < 0.01, *** indicates P < 0.001.
